# Maternal circulating leukocytes display early chemotactic responsiveness during late gestation

**DOI:** 10.1186/1471-2393-13-S1-S8

**Published:** 2013-01-31

**Authors:** Nardhy Gomez-Lopez, Satomi Tanaka, Zoya Zaeem, Gerlinde A Metz, David M Olson

**Affiliations:** 1 Departments of Obstetrics and Gynecology, Pediatrics and Physiology, University of Alberta, Edmonton, T6G 2S2, Canada; 2 Department of Obstetrics and Gynecology, School of Medicine, Perinatology Research Branch, Wayne State University, Detroit, MI, 48201, USA; 3 Canadian Centre for Behavioral Neuroscience, University of Lethbridge, Lethbridge, T1K3M4, Canada

## Abstract

**Background:**

Parturition has been widely described as an immunological response; however, it is unknown how this is triggered. We hypothesized that an early event in parturition is an increased responsiveness of peripheral leukocytes to chemotactic stimuli expressed by reproductive tissues, and this precedes expression of tissue chemotactic activity, uterine activation and the systemic progesterone/estradiol shift.

**Methods:**

Tissues and blood were collected from pregnant Long-Evans rats on gestational days (GD) 17, 20 and 22 (term gestation). We employed a validated Boyden chamber assay, flow cytometry, quantitative real time-polymerase chain reaction, and enzyme-linked immunosorbent assays.

**Results:**

We found that GD20 maternal peripheral leukocytes migrated more than those from GD17 when these were tested with GD22 uterus and cervix extracts. Leukocytes on GD20 also displayed a significant increase in chemokine (C-C motif) ligand 2 (*Ccl2*) gene expression and this correlated with an increase in peripheral granulocyte proportions and a decrease in B cell and monocyte proportions. Tissue chemotactic activity and specific chemokines (CCL2, chemokine (C-X-C motif) ligand 1/CXCL1, and CXCL10) were mostly unchanged from GD17 to GD20 and increased only on GD22. CXCL10 peaked on GD20 in cervical tissues. As expected, prostaglandin F2α receptor and oxytocin receptor gene expression increased dramatically between GD20 and 22. Progesterone concentrations fell and estradiol-17β concentrations increased in peripheral serum, cervical and uterine tissue extracts between GD20 and 22.

**Conclusion:**

Maternal circulating leukocytes display early chemotactic responsiveness, which leads to their infiltration into the uterus where they may participate in the process of parturition.

## Background

Parturition resembles an immunological response, which is characterized by the infiltration of immunological cells and secretion of immunological mediators (i.e. cytokines) in the maternal-fetal tissues [1-3]. A significant role for this immunological response in most (if not all) labors has been confirmed, regardless of the presence of infection or timing of delivery [4-6]. Given that inflammatory processes seem to activate parturition and are intertwined with early (or upstream), mid (or midstream) and late (or downstream) phases of the birth cascade, there are many research groups, including our group, focused on understanding how they trigger the termination of gestation. This knowledge will reveal mechanisms and possibly prevent pregnancy complications such as preterm birth.

Accordingly, it is essential to understand how this inflammatory stage is created in the maternal-fetal tissues. Leukocyte recruitment appears as the first step in the conditioning of this stage [7]. There is evidence that activated leukocytes extravasate from the local circulation into these tissues [8,9] by chemotactic processes and expression of specific chemokines [10-12]. Several chemokines have been studied in the maternal fetal tissues. Among the most relevant we will mention three of them: chemokine (C-C motif) ligand 2 (CCL2) or monocyte chemotactic protein-1 (MCP-1) that participates in the monocyte recruitment, chemokine (C-X-C motif) ligand 1 (CXCL1) or neutrophil-activating protein 3 (NAP-3) that participates in the neutrophil recruitment, and CXCL10 or interferon gamma-induced protein 10 (IP-10) that participates in the T-cell recruitment [7,11]. Infiltrated leukocytes create this particular inflammatory stage, and along with maternal-fetal tissues, secrete other inflammatory mediators, including pro-inflammatory cytokines, prostaglandins and corticotrophin releasing hormone, that stimulate expression of uterine activation proteins (UAPs) [13]. There are at least nine UAPs [14] and likely many more, including matrix metalloproteinases (MMPs) that are particularly important in converting the uterus of pregnancy to the uterus of labor [15,16]. These actions are feed-forward and iterative thereby amplifying the inflammatory stage and birth cascade, which leads to the downstream events of parturition: membrane rupture, cervical dilatation, myometrial contractility, placental separation, and uterine involution [17]. Concomitantly, the influence of progesterone (P4) in maintaining pregnancy and suppressing inflammation wanes and estradiol-17β (E2) levels rise [18,19], thereby permitting and accelerating the changes leading to parturition.

The purpose of this initial, observational study was to clarify the timing of key relationships in the birth cascade to one another in a rat model. We hypothesized that an early event in parturition is due to an increased responsiveness in peripheral leukocytes to chemotactic stimuli expressed by the gestational tissues. This event precedes the expression of UAPs and the systemic progesterone/estradiol shift in rodents.

## Methods

### Animals

Animal protocols were approved by the University of Alberta Health Sciences Animal Policy and Welfare Committee (#625/03/11/C), and the experiments were conducted in accordance with the Guidelines and Policies of Canadian Council on Animal Care (SOP.RES:RA:0001.0). Timed-pregnant Long-Evans rats weighing 250-350 g were obtained from Charles River (Sherbrooke, PQ) on gestational day (GD) 11-12. Rats were housed in the University of Alberta Health Sciences Laboratory Animal Services.

### Tissues and blood

Rats were euthanized according to approved local policy with a lethal dose of isoflurane (Halocarbon Products Corporation, USA) by inhalation in a large beaker on GD17, 20 or 22 (n=5 each). Parturition occurs at GD22.5. Blood by heart puncture (10 mL) and maternal-fetal tissues were collected in sterile conditions from post-mortem rats. Maternal-fetal tissues included uterus, cervix, fetal membranes and placenta. Tissues were washed in saline solution and placed immediately in RNALater (Ambion, Applied Biosystems, USA) or in liquid nitrogen (and then kept at -80ºC) until they were assayed.

### Leukocytes and serum

Blood samples were immediately divided into two tubes: 5 mL into a heparinized vacuum tube (BD Vacutainer, USA) and 5 mL into a 15 mL centrifuge tube (Corning Incorporated, USA). Serum was isolated from the centrifuge tube and stored at -20ºC until assayed. Polymorphonuclear and mononuclear leukocytes were isolated from the heparinized tube using a Ficoll gradient (Polymorphoprep, Axis-Shield, Norton, USA) following the manufacturer’s instructions. Total leukocytes were then washed in 1X PBS and the pellet was resuspended and stored in 500 µL of RNALater or resuspended and incubated in supplemented RPMI medium 1640 (1% antibiotics [5,000 units of penicillin and 5,000 µg of streptomycin/mL] and 10% fetal bovine serum) (Invitrogen, USA) at 37ºC for 24 h. Following the incubation time these leukocytes were used in chemotaxis assays.

### Protein extracts of tissues

Tissues previously placed in liquid nitrogen were gradually defrosted. Tissues were cut in fragments of ~1-cm diameter and homogenized in 1 mL of DMEM (High Glucose 1X and 1% antibiotics) (Invitrogen) using a Polytron (Polytron PRO200 homogenizer, PRO Scientific, USA). These extracts were centrifuged at 4ºC, 12,000 x *g* for 30 min, repeating this last step until a clear supernatant was obtained. Protein extracts then were stored at -20ºC until use.

### Chemotaxis assay

The chemotaxis assay was performed using a modified validated Boyden chamber assay (AP48, Neuro Probe, USA) [20]. First, we investigated the onset of peripheral leukocyte migration or chemotaxis during gestation in response to uterine and cervical extracts obtained at term (GD22). Chemotaxis assays were performed using extracts from uterine and cervical tissues obtained on GD22 and maternal peripheral leukocytes obtained on GD17, 20 and 22. Second, we tested the migratory activity of leukocytes at term gestation (GD22) against uterine, cervical, fetal membrane and placental extracts obtained from GD17, 20 and 22. According to the above, 50 μL of leukocyte suspension containing 10^5^ leukocytes was placed on top of the polycarbonate membrane (5µm pore size, #PFB5, Neuro Probe) with either extracts from uterine or cervical tissues as chemoattractant in the lower compartment. Chambers were incubated for 120 min at 37°C in humidified air containing 5% CO_2_. Incubation time was previously established by performing incubation time curves [20]. Afterwards, chemoattracted leukocytes were removed from the lower compartment and centrifuged at 500 × *g* for 5 min at room temperature. The pellet was fixed with 500 µL of OptiLyse® B Lysing Solution (Beckman Coulter, USA) and counted by flow cytometry. The flow cytometer (FACSCANTO II, http://flowcytometry.med.ualberta.ca/ ) was set to analyze the samples for 30 s, which involved 10^4^–10^5^ events. The coefficient of variance of this method (inter and intra assay) is < 5%. Leukocytes attracted by the negative control (DMEM medium) were subtracted in all cases.

### Flow cytometry

Blood taken by heart puncture was labeled with the following fluorochrome-conjugated anti-rat mAb: CD45-Alexa Fluor 488 for total leukocytes (clone OX-1, #202205, BioLegend, USA), OX43-PE for monocytes (sc-53109, Santa Cruz Biotechnology, USA), CD45R-PE for B cells (clone HIS24, #554881, BD Pharmingen, USA), CD161-Alexa Fluor 647 for NK cells (10/78, #203110, BioLegend) and CD3-FITC/CD4-PC7/CD8-APC for T cells (clones 1F4, OX-38, OX-8, #PN A32909; Beckman Coulter). Additionally, we identified mast cells using a primary purified mouse anti-rat Mast cell (clone AR32AA4, #551770, BD Pharmigen) and a secondary rabbit anti-mouse IgG_2a_-PE (sc-3765, Santa Cruz Biotechnology). Leukocytes were then fixed using 500 µL of OptiLyse B (Beckman Coulter), washed, and resuspended in 500 µL of 1X PBS to be analyzed by flow cytometry. The flow cytometer was set to analyze 10^5^ events. The phenotype of leukocytes was analyzed within the CD45^+^ and CD3^+^ region, respectively. Granulocytes were identified as CD45^+^OX43^-^CD45R^-^CD161^-^CD3^-^ by using the parameters of FS, SS and CD45 [20].

### RNA extraction, complementary DNA (cDNA) synthesis and quantitative real time-polymerase chain reaction (qPCR)

RNAlater was removed from the tissue samples and leukocytes by aspiration or centrifugation, respectively. Total RNA was isolated from them using Trizol (Invitrogen) following the manufacturer’s instructions. Total RNA concentration was quantified using the spectrophotometer ND-1000 (Thermo Fisher Scientific Inc, USA). Total RNA concentration was determined with spectrophotometer at 260 nm optical density. The RNA purity was assessed by the optical density ratio 260 nm/280 nm (~2.0). cDNA was synthesized from 500 ng of total RNA using the qScript™ cDNA SuperMix (Quanta BioSciences, USA), following manufacturer’s instructions. qPCR was performed using the SYBR Green FastMix (Quanta BioScience) and the iCycler apparatus (Bio-Rad, USA). Primers for rat chemokines and UAPs were designed using the Primer Premier 5 software (Table 1). The cDNA obtained was then used in subsequent qPCR reactions. Each reaction contained 1 μL of cDNA (50 ng/µL), 10 μL of SYBR green FastMix, 0.5 μL of forward primer (10 µM), 0.5 μL of reverse primer (10µM) and sterile water in a total reaction volume of 20 μL. qPCR was performed under the following conditions: 10 min at 95 ºC, followed by 40 cycles of 15 s at 95 ºC and 1 min at 60-62 ºC (Table 1). To control for amplification of non-specific products, melt curve analysis was performed following amplification by measuring fluorescence while increasing temperature in 0.5 ºC increments from 55 ºC to 95 ºC. No amplification of non-specific products was observed with each set of primers. Standard curves for each gene were generated by serial dilutions of pooled cDNA samples. The amplification efficiency for each primer set was determined by converting the slope of the standard curve using the algorithm E = 10^–1/slope^. For each gene, the mean threshold cycle (from duplicate reactions) was corrected for the efficiency of the reaction and expressed relative to a control sample for each experiment. Rat chemokine and UAP levels were then expressed relative to cyclophilin A (*Cyp*) levels, the housekeeping gene [21].

**Table 1:** Primers used for RT-PCR

Gene	Primers	**Optimal Temperature (**ºC**)**	NCBI^a^ Reference Sequences
***Cxcl1***	f: 5’-gca CCC AAA CCG AAG TCA-3’ r: 5’-AAG CCA GCG TTC ACC AGA-3’	60	NM_030845
***Cxcl10***	f: 5’- GGT GAG CCA AAG AAG GTC-3’ r: 5’-ACA CTG GGT AAA GGG AGG-3’	60	NM_139089.1
***Ccl2***	f: 5’-GCA GGT GTC CCA AAG AAG-3’ r: 5’-TCA AAG GTC CTG AAG TCC-3’	60	NM_031530
***Fp***	f: 5’-CTGGCCATAATGTGCGTCTC-3’ r: 5’-TGTCGTTTCACAGGTCACTGG-3’	60	NM-013115.1
***Otr***	f: 5’-CGATTGCTGGGCGGTCTT-3’ r: 5’-CCGCCGCTGCCGTCTTGA-3’	62	NM-012871.2
***Cyp***	f: 5’-CACCGTGTTCTTCGACATCAC-3’ r: 5’-CCAGTGCTCAGAGCTCGAAAG-3’	60	NM-017101

^a^http://www.ncbi.nlm.nih.gov

### Enzyme-linked immunosorbent assays (ELISAs)

ELISAs for rat CXCL1 (#RCN100, R&D Systems, USA; sensitivity = 0.7 - 1.3 pg/mL; Intra- and inter-assay precision = 6 and 5.7%), CXCL10 (#E90371Ra, USCN Life Science, USA; sensitivity < 12.5 pg/mL; Intra- and inter-assay precision = 10 and 12%) and CCL2 (#KRC1011, Invitrogen; sensitivity < 8 pg/mL; Intra- and inter-assay precision = 5.7 and 8.4%) were performed in protein extracts of tissues following manufacturer’s instructions. ELISAs for P4 (#PG129S-100, Calbiotech, USA; sensitivity < 0.22 ng/ml; Intra- and inter-assay precision = 2.6 and 8.2 %) and E2 (#ES180S-100, Calbiotech; sensitivity < 3.94 pg/ml; Intra- and inter-assay precision = 8.1 and 5.8%) were performed in both serum and protein extracts of tissues following the manufacturer’s instructions. The protein concentration of protein extracts were measured with Protein Assay Reagent (Precision Red^TM^, Cytoskelton, USA) at UV-vis 600 nm by the ND-1000. Chemokine and hormonal concentrations were normalized with protein concentrations.

### Statistical analyses

The data were examined initially by the Shapiro-Wilk test for normal distribution. The Kruskal-Wallis and Mann–Whitney U tests were used since the data were not normally distributed. Statistical analyses were performed using SPSS (SPSS Inc, USA), version 18.0. A P value of ≤ 0.05 was considered statistically significant.

## Results

### Leukocyte migration - chemotactic responsiveness

**Figure 1:**
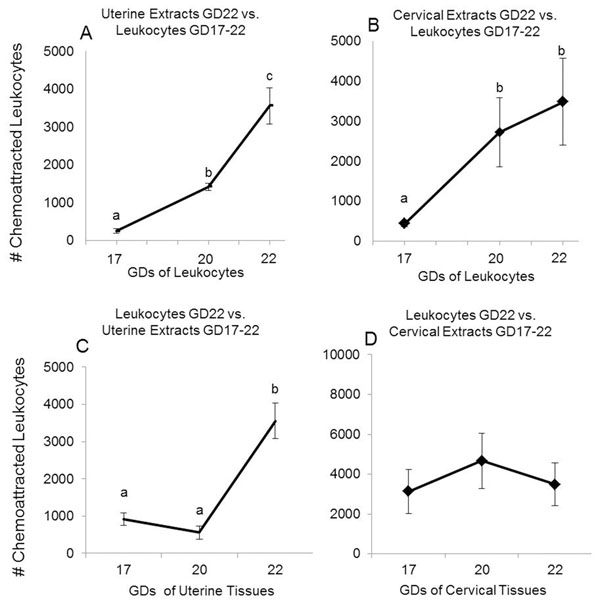
Leukocyte migration - chemotactic responsiveness. Maternal peripheral leukocytes from GD17, 20 and 22 were tested in chemotaxis assays using extracts from uterine segments (A) and cervix (B) obtained on GD22. Uterine (C) and cervical (D) protein extracts of tissues on GD17, 20 and 22 were tested with leukocytes on GD22. Data are presented as mean ± SEM of attracted leukocytes in triplicate by each group of tissues (n=5 each). Means with different letters are significantly different. P values: A) a vs. b p<0.0001, b vs. c p=0.024, a vs. c p=0.005; B) a vs. b p<0.05; C) a vs. b p< 0.009.

First, we investigated the onset of peripheral leukocyte migration or chemotaxis during gestation in response to uterine and cervical extracts obtained at term. Chemotaxis assays were performed using extracts from uterine and cervical tissues obtained on GD22 and maternal peripheral leukocytes obtained on GD17, 20 and 22. Our data show clearly that there is an increase in leukocyte migration on GD20 compared to GD17 in response to term chemotactic signals from the uterus and the cervix (p<0.05, figure 1A, B). This high leukocyte responsiveness was increased further at GD22 (p=0.024) in response to chemotactic factors extracted from the term uterus (figure 1A). In contrast, when the leukocytes were obtained on GD22 and their migratory activity was tested against uterine or cervical chemotactic factors obtained from GD17-22, there was an increase in chemotactic activity only on GD22 in the uterus (figure 1C) and cervical chemotactic activity was similar on each day (figure 1D). Extracts from placenta and fetal membranes did not show differences in leukocyte chemotactic activity between GDs (data not shown). These data suggest that the sensitivity of peripheral leukocytes to chemotactic signals from uterine or cervical tissues is dynamic and is changing more rapidly in the final days of gestation compared to the production of chemotactic signals by these tissues. We then queried the temporal relationship of the increased responsiveness between GD17 and 20 with other events associated with the birth cascade.

### Expression of chemokines in maternal circulating leukocytes

**Figure 2:**
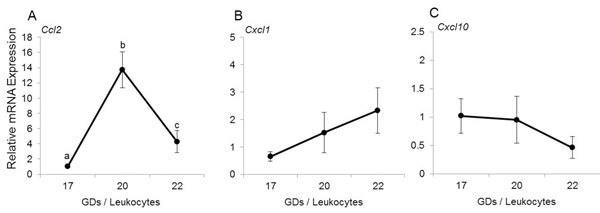
Chemokine expression in maternal peripheral leukocytes. Relative mRNA expression of *Ccl2* (A), *Cxcl1* (B) and *Cxcl10* (C) in total peripheral leukocyte population. Data shown are means ± SEM of determinations in duplicate per group (n = 5 each). Means with different letters are significantly different. P values: A) a vs. b p=0.016, b vs. c p=0.032, a vs. c p=0.016

Next, we ascertained whether this high leukocyte responsiveness on GD20-22 was associated with expression of 3 chemokines: CXCL1, CXCL10 and CCL2 (figure 2). It is evident that *Ccl2* expression in circulating leukocytes is increased significantly (p<0.016, figure 2A) between GD17 and 20, then decreases but remains higher on GD22 than on GD17. Neither *Cxcl1* nor *Cxcl10* significantly changed (figure 2B, C, not significant).

### Leukocyte subsets - maternal circulation

**Figure 3:**
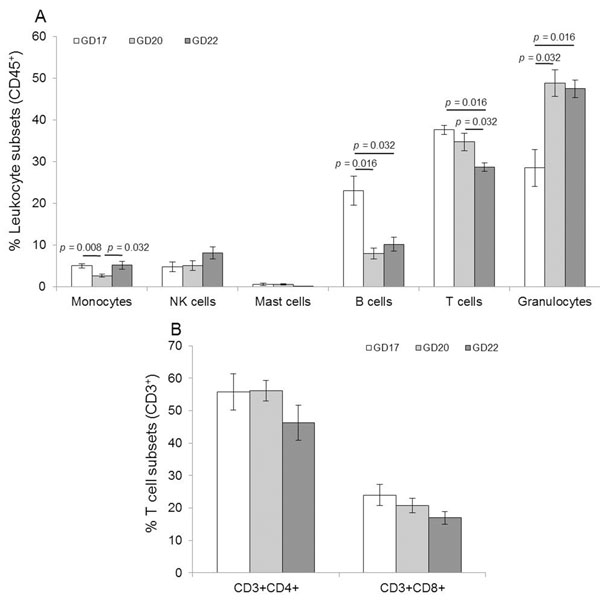
Leukocyte subsets - maternal peripheral circulation. Leukocyte phenotype was determined by using monoclonal conjugated fluorochromes and flow cytometry. A, Total Leukocytes. B, T cell subsets. Data are presented as mean ± SEM of determinations in duplicate per group of tissues (n=5 each).

An event that coincides with the end of gestation and beginning of parturition is the changing of leukocyte subsets in maternal peripheral circulation [22]. We found significant changes in the proportions of some leukocyte subsets in the maternal periphery (figure 3). Monocytes were lower on GD20 (*p*=0.008) than on the other days. B cells decreased by more than half from GD17 to 20 (*p*=0.016), and their proportions remained stable. Between GD20 and GD22 T cells decreased (*p*=0.032 and 0.016, relative to GD20 and 17, respectively), while in contrast granulocytes nearly doubled (*p*=0.016). There were no significant changes in late gestation in CD3^+^CD4^+^ cells or CD3^+^CD8^+^ cells.

### Expression of chemokines in the maternal-fetal tissues

While maternal-fetal tissue leukocyte chemotaxis represents the dynamic relationship between total tissue chemotactic capacity and overall leukocyte responsiveness, it is of considerable interest to examine the expression of individual chemokines in several tissues such as cervix, uterus, fetal membranes and placenta during late rat gestation. We therefore examined the mRNA and protein expression of CCL2, chemokine for monocytes, CXCL1 for neutrophils, and CXCL10 for T cells.

#### *Ccl2*/CCL2

**Figure 4:**
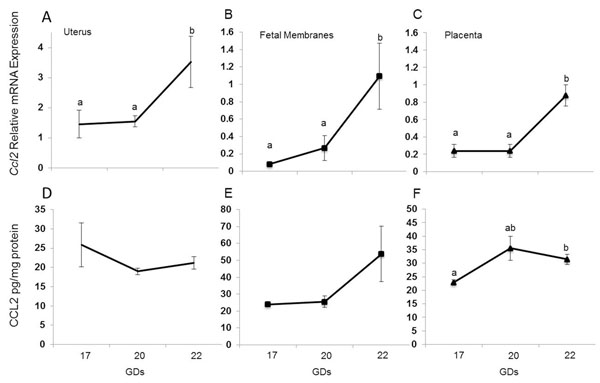
*Ccl2*/CCL2 in gestational tissues. Relative mRNA expression and concentration of *Ccl2/*CCL2 in uterus (A and D), fetal membranes (B and E) and placenta (C and F). Data are presented as mean ± SEM of determinations in duplicate per group of tissues (n=5 each). Means with different letters are significantly different. P values (a vs. b) : A) p=0.04; B) p=0.022; C) p= 0.008; F) p=0.040.

*Ccl2* levels did not change between GD17 and 20, but they did increase from GD20 to 22 such that they were significantly higher on GD22 than 20 in the uterus (*p*=0.042, figure 4A), in fetal membranes (*p*=0.022, figure 4B), and in placenta (*p*=0.008, figure 4C). Unlike the *Ccl2* levels, the CCL2 concentrations in the uterus did not change between gestational days (figure 4D); however, they displayed an increasing trend from GD20 to 22 in the fetal membranes (figure 4E) and from GD17 to 22 in placenta (*p*=0.040, figure 4F). CCL2 mRNA abundance and protein in cervix did not change between gestational days (data not shown).

#### *Cxcl1*/CXCL1

**Figure 5:**
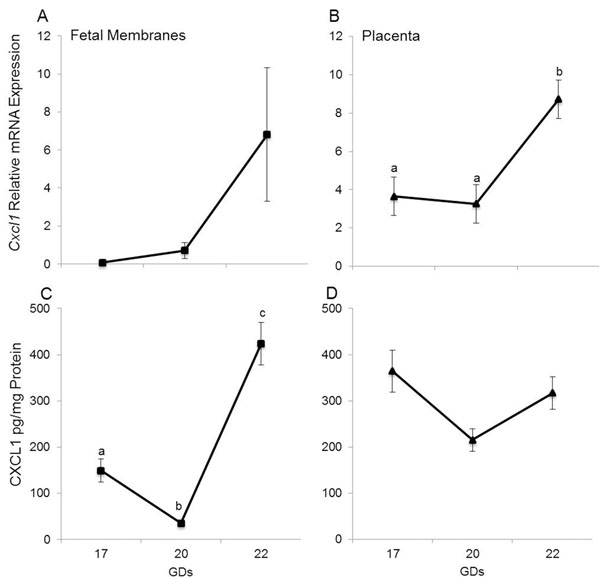
*Cxcl1*/CXCL1 in fetal membranes and placenta. Relative mRNA expression and concentration of *Cxcl1/*CXCL1 in fetal membranes (A and C) and placenta (B and D). Data are presented as mean ± SEM of determinations in duplicate per group of tissues (n=5 each). Means with different letters are significantly different. P values: B) a vs. b p<0.028; C) a vs. b p<0.044, b vs. c p=0.009, a vs. c p=0.02.

*Cxcl1* mRNA and CXCL1 protein abundance in the uterus and cervix did not change between groups (data not shown). But *Cxcl1* and CXCL1 expression trended towards increasing from GD20 to 22 in the fetal membranes (*Cxcl1* not significant, CXCL1 *p*=0.009, figure 5A,C) and placenta (*Cxcl1 p*=0.028, CXCL1 not significant) (figure 5B&D). Curiously, fetal membrane CXCL1 levels decreased from GD17 to 20 (*p*=0.044) (figure 5C).

#### *Cxcl10*/CXCL10

**Figure 6:**
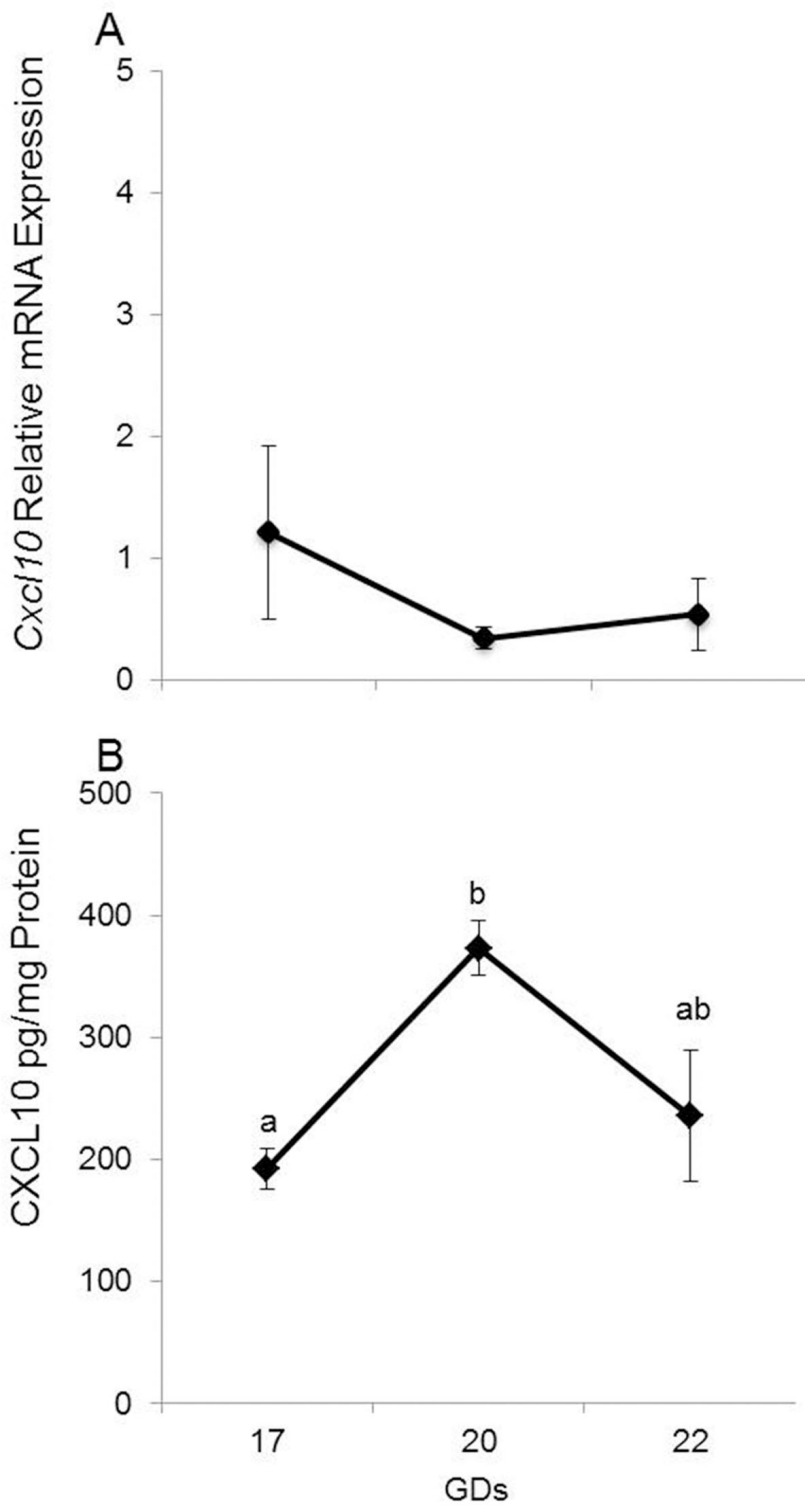
*Cxcl10*/CXCL10 in cervix. Relative mRNA expression (A) and concentration (B) of *Cxcl10*/CXCL10 in cervix Data are presented as mean ± SEM of determinations in duplicate per group of tissues (n=5 each). Means with different letters are significantly different. P values: B) a vs. b p<0.02.

Although *cxcl10* mRNA did not change between GDs, cervical CXCL10 levels peaked on GD20 (*p*=0.02, figure 6). CXCL10 mRNA and protein abundance did not change between gestational days in other tissues (data not shown).

### P4 and E2 concentrations in serum and tissue extracts

**Figure 7:**
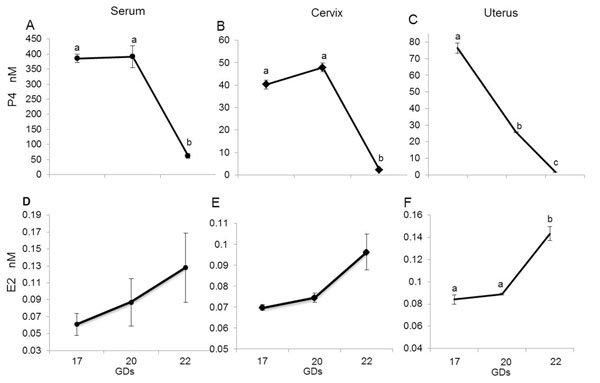
P4 and E2 concentrations. P4 and E2 concentrations in serum (A and D), cervix (B and E) and uterus (C and F). Data shown are means ± SEM of determinations in duplicate per group (n= 5 each). Means with different letters are significantly different. P values: A) a vs. b p<0.0001; B) a vs. b p=0.002; C) a vs. b p=0.042, b vs. c p=0.014, a vs. c p=0.001; F) a vs. b p=0.016.

We observed the classical shift in the steroid hormone concentrations as evidenced by a P4 fall and E2 rise, which occurred between GD20 and 22 in peripheral serum, cervix and uterus for P4 (*p*≤0.014; figure 7A-C). Conversely, E2 levels tended to increase from GD20 to 22, but this increase was significant only in uterine extracts (p=0.016) (figure 7D-F).

### Maternal uterine activation - UAP expression

**Figure 8:**
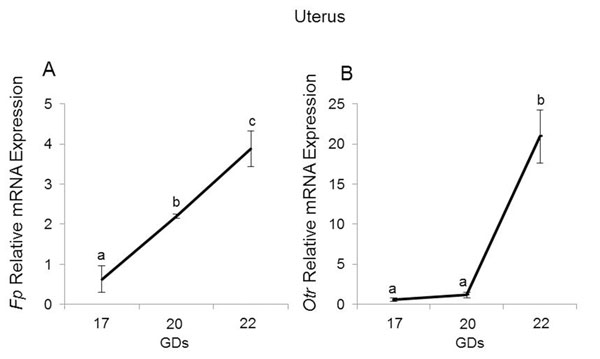
Maternal uterine activation - UAP expression. Relative mRNA expression of *Fp* (A) and *Otr* (B) in uterus. Data shown are means ± SEM of determinations in duplicate per group (n= 5 each). Means with different letters are significantly different. P values: A) a vs. b p=0.045, b vs. c p=0.05, a vs. c p=0.02; B) a vs. b p=0.0257.

Last, we correlated these immunological and steroid hormone changes with the expression of two key UAPs, prostaglandin F2α receptor (*Fp*) and oxytocin receptor (*Otr*), which are ultimately responsible for preparing the uterus for labor and delivery. In the uterus, *Fp* levels increased gradually from GD17 to 22 (p<0.05, figure 8A). Uterine *Otr* levels also increased from GD20 to 22 (p=0.027, respectively; figure 8B).

## Discussion

The data of this study demonstrate that maternal peripheral leukocytes display some of the earliest changes (by GD20) that precede parturition in the Long-Evans rat. The most telling of these is their ability to increase their migration through a Boyden chamber in response to a term uterine or cervical chemotactic stimulus. Next is the ability to increase their expression of *Ccl2*, and early changes in the relative proportions of monocytes, B cells and granulocytes. Later, between GD20 and 22, further changes occur in leukocytes, serum, uterus, cervix, fetal membranes and placenta. These include increased activity of chemotactic factors, changes in tissue and circulating levels of steroids, and the up-regulation of UAPs. These data suggest that an increased responsiveness of maternal peripheral leukocytes may be an early step in the birth cascade. Subsequent steps could include the invasion of uterine tissues by responsive leukocytes where they contribute to creating local inflammatory microenvironments that promote parturition through stimulation of chemokines and chemokine activity and the stimulation of UAP expression.

Long-Evans rats may be an appropriate model for immunological studies such as exploring the relationships between leukocyte migration responsiveness, chemokine activity, and uterine activation. Previous studies have demonstrated that Long-Evans rats alter their gestation length when treated with pro-inflammatory agents [23], whilst other rat strains such as the Sprague-Dawley does not appear to respond accordingly [24]. Another advantage over the Sprague-Dawley rat model is that the Long-Evans dam is 50% or more larger, thereby facilitating collection of blood and tissues.

The Boyden chamber migration assay is a very sensitive technique for assessing leukocyte responsiveness to chemotactic signals [20]. To our knowledge, this is the first time it has been used in an animal study with the purpose of delineating temporal events in the birth cascade. Previously, we used it to demonstrate that the human maternal-fetal interface, fetal membranes, and choriodecidua exhibit chemotaxis of maternal peripheral leukocytes and this increases during term labor [10,12]. This chemotaxis correlated with specific chemokine expression and infiltration of neutrophils, macrophages and T cells [7,11]. Here we used term chemotactic activity extracted from various tissues and tested it against leukocytes collected at different times in late gestation to demonstrate the changing responsiveness of leukocytes to the same chemotactic signals as gestation progressed.

First, we demonstrated that before term (GD20) maternal circulating leukocytes expressed very high levels of *Ccl2*, which correlates with their high responsiveness to be attracted by the uterine and cervical extracts. Increased migratory responsiveness is an indication of increased leukocyte activation as activated leukocytes often constitutively express high-affinity forms of integrins and chemokine receptors, which increase their ability to migrate in response to chemokines [25,26]. Further studies will be required to prove that they are actually activated and therefore invade the uterus. The important message is that the timing of the increased migratory responsiveness occurs before term pregnancy, which means that this event precedes parturition.

CCL2 is produced by many cell types and is a potent chemoattractant for monocytes and basophils that express its receptors, CCR2 and CCR4. Besides acting as chemokine, CCL2 induces the expression of integrins required for chemotaxis [27]. We suggest that *Ccl2* is expressed by maternal circulating responsive leukocytes to facilitate the recruitment of monocytes and other leukocytes expressing its receptors into the reproductive tissues (e.g. uterus and cervix). In addition, our data showed that the proportion of monocytes in the maternal circulating blood decreases at this time. This last may mean that monocytes are recruited massively towards these tissues and therefore their proportions decrease in the maternal peripheral blood before term pregnancy.

The uterus and the cervix are interesting not only in their respective roles in parturition (contraction vs. softening and effacement), but each produced term chemotactic activity that attracted more leukocytes on GD20 than GD17. This suggests that these tissues also play a chemotactic role during late gestation.

Our data show that the uterus at term exhibits increased leukocyte chemotaxis and this coincides with high expression of *Ccl2*. CCL2 is largely identified with the recruitment of monocytes into the uterus and its levels increase throughout late gestation [28]. Because our results showed that the protein levels of CCL2 do not increase on term pregnancy, we suggest that other chemokines (and other chemotactic factors) may be participating in this increased leukocyte chemotaxis that we observed. Future studies will perform protein microarrays in uterine extracts to investigate which chemotactic factors participate in this event during term pregnancy.

Although mRNA levels of *Ccl2* and *Cxcl1* in placenta and fetal membranes tended to increase at term gestation, these tissue extracts did not contain the protein nor exhibit differential chemotactic activity throughout late gestation (data not shown). This finding suggests that these tissues can express these chemokines and therefore they contribute in the creation of chemotactic gradients that promote leukocyte infiltration into the maternal-fetal interface (i.e. uterus and decidua); however, unlike the uterus they do not participate in the active protein-related chemotaxis of leukocytes. Besides contributing to *Cxcl1* expression, fetal membranes may also contain external chemokines (e.g. uterine chemokines) because these tissues have a high content of extracellular matrix, and it has been established that chemokines have high affinity for extracellular matrix proteins [29].

Indeed, the level of cervical chemotactic activity far exceeded that from any other uterine tissue. Our data also showed that the cervix has a peak of CXCL10 on GD20. CXCL10 helps to recruit effector T cells [30], and it is also produced by granulocytes in an extremely pro-inflammatory microenvironment [31](e.g. labor). Granulocytes, monocytes and some T cells infiltrate the human cervix during late gestation [32]. Monocytes seem to participate in the cervical remodeling before the onset of labor [33], granulocytes seem to play a key role at the postpartum period [34,35]; however, the T cell function is unclear. Recently, we demonstrated that T cells infiltrate the cervical zone of the fetal membranes during normal labor and that these cells are associated with MMP-9 [36,37], which also participates in surrounding tissue remodeling [38]. We suggest that CXCL10 on GD20 may participate in the recruitment of T cells into the cervix, which could participate in the cervical softening before term pregnancy [39].

The fall in tissue and circulating P4 concentrations and the rise in E2 concentrations is expected and occurs as a consequence of luteolysis. P4 withdrawal contributes to the recruitment of immune cells into the rat uterus and cervix [40], and it is associated with the increase in UAPs, especially OTR and FP [14,41].

Currently, we cannot explain the discrepancies between the mRNA levels/protein levels of chemokines and chemotactic activity in the maternal-fetal tissues. We can only speculate that chemotactic factors released by specific reproductive tissues may participate in the leukocyte recruitment into the maternal-fetal interface. However, given the specific properties of uterine chemotactic factors in contrast to those from other tissues in terms of the graded and increasing attraction of peripheral leukocytes as pregnancy wanes, it is obvious that this is a complicated area requiring considerable further study. Characterizing and mechanistic studies are needed to identify all key chemokines that exist, their specific receptors and the precise cellular adhesion molecules that actively participate in these events during pregnancy and term labor.

## Conclusion

In conclusion we demonstrated that the maternal circulating leukocytes increase their responsiveness to migrate to term chemotactic stimuli, and this coincides with their high expression of *Ccl2*, and changes in relative proportions in peripheral blood. These events precede uterine tissue chemotactic activity expression, expression UAPs, and the changes in steroid hormone concentrations typical of the termination of pregnancy. We surmise that peripheral leukocyte responsiveness precedes leukocyte recruitment to specific gestational tissues, including the cervix and uterus [42]. These create a tissue specific immunological microenvironment that promotes tissue activation leading to labor at term of pregnancy. Knowing these events in rats and other animal models and eventually translating these outcomes to women, will assist in identifying points of convergence of the birth cascade and new therapeutic targets to treat problems such as preterm delivery.

## List of abbreviations used

CCL2: Chemokine (C-C motif) ligand 2*; CD: Cluster of differentiation; CXCL1: Chemokine (C-X-C motif) ligand 1*; CXCL10: Chemokine (C-X-C motif) ligand 10*; Cyp: Cyclophilin A*; DMEM: Dulbecco's Modified Eagle Medium; E2: Estradiol-17β; ELISA: Enzyme-linked immunosorbent assay; Fp: Prostaglandin F receptor*; GD: Gestational days; IP-10: Interferon gamma-induced protein 10; MCP-1: monocyte chemotactic protein-1; MMP-9: Matrix metallopeptidase 9; NAP-3: Neutrophil-activating protein 3; Otr: Oxytocin receptor*; P4: Progesterone; qPCR: Quantitative real time-polymerase chain reaction; UAPs: Uterine activation Proteins

*Lower case italics were used for gene names and upper case normal fonts were used for protein names

## Competing interests

Authors have no conflicts of interest to declare

## Authors’ contributions

NG-L, ST and DMO: Conception, design of the experiments and drafting the article. All authors: Collection, analysis and/or interpretation of data, revision and approbation of the final version of the article.

## Declarations

This article has been published as part of *BMC Pregnancy and Childbirth* Volume 13 Supplement 1, 2013: Preterm Birth: Interdisciplinary Research from the Preterm Birth and Healthy Outcomes Team (PreHOT). The full contents of the supplement are available online at http://www.biomedcentral.com/bmcpregnancychildbirth/supplements/13/S1.

All of the publication fees will be funded by the Preterm Birth and Healthy Outcomes Team Interdisciplinary Team Grant (#200700595) from Alberta Innovates - Health Solutions, formerly the Alberta Heritage Foundation for Medical Research.
